# Surfactant-Mediated Structural Modulations to Planar, Amphiphilic Multilamellar Stacks

**DOI:** 10.1021/acs.jpcb.3c01654

**Published:** 2023-08-16

**Authors:** Daniel J. Speer, Marta Salvador-Castell, Yuqi Huang, Gang-Yu Liu, Sunil K. Sinha, Atul N. Parikh

**Affiliations:** † Chemistry Graduate Group, University of California, Davis, One Shields Avenue, Davis, California 95616, United States; ‡ Department of Physics, University of California, San Diego, 9500 Gilman Drive, La Jolla, California 92093, United States; § Department of Chemistry, University of California, Davis, One Shields Avenue, Davis, California 95616, United States; ∥ Department of Biomedical Engineering, University of California, Davis, One Shields Avenue, Davis, California 95616, United States

## Abstract

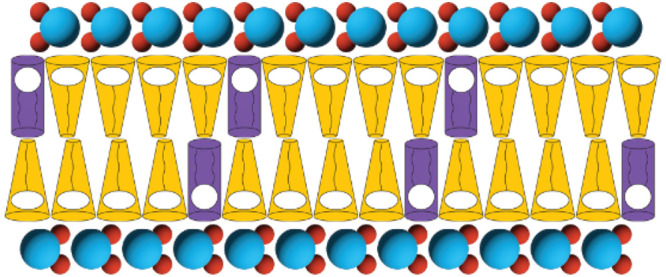

The hydrophobic effect, a ubiquitous process in biology, is a primary thermodynamic driver of amphiphilic self-assembly. It leads to the formation of unique morphologies including two highly important classes of lamellar and micellar mesophases. The interactions between these two types of structures and their involved components have garnered significant interest because of their importance in key biochemical technologies related to the isolation, purification, and reconstitution of membrane proteins. This work investigates the structural organization of mixtures of the lamellar-forming phospholipid 1-palmitoyl-2-oleoyl-*sn*-glycero-3-phosphocholine (POPC) and two zwitterionic micelle-forming surfactants, being *n*-dodecyl-*N*,*N*-dimethyl-3-ammonio-1-propanesulfonate (Zwittergent 3-12 or DDAPS) and 1-oleoyl-2-hydroxy-*sn*-glycero-3-phosphocholine (O-Lyso-PC), when assembled by water vapor hydration with X-ray diffraction measurements, brightfield optical microscopy, wide-field fluorescence microscopy, and atomic force microscopy. The results reveal that multilamellar mesophases of these mixtures can be assembled across a wide range of POPC to surfactant (POPC:surfactant) concentration ratios, including ratios far surpassing the classical detergent-saturation limit of POPC bilayers without significant morphological disruptions to the lamellar motif. The mixed mesophases generally decreased in lamellar spacing (*D*) and headgroup-to-headgroup distance (*D*_hh_) with a higher concentration of the doped surfactant, but trends in water layer thickness (*D*_w_) between each bilayer in the stack are highly variable. Further structural characteristics including mesophase topography, bilayer thickness, and lamellar rupture force were revealed by atomic force microscopy (AFM), exhibiting homogeneous multilamellar stacks with no significant physical differences with changes in the surfactant concentration within the mesophases. Taken together, the outcomes present the assembly of unanticipated and highly unique mixed mesophases with varied structural trends from the involved surfactant and lipidic components. Modulations in their structural properties can be attributed to the surfactant’s chemical specificity in relation to POPC, such as the headgroup hydration and the hydrophobic chain tail mismatch. Taken together, our results illustrate how specific chemical complexities of surfactant–lipid interactions can alter the morphologies of mixed mesophases and thereby alter the kinetic pathways by which surfactants dissolve lipid mesophases in bulk aqueous solutions.

## Introduction

The hydrophobic interaction—the water-induced attraction between nonpolar molecules (or parts thereof)—is a primary driving force for the spontaneous self-assembly of amphiphilic lipids in water.^1^ Together with the molecular packing characteristics, this hydrophobic effect gives rise to a rich phase behavior, stabilizing a variety of well-ordered lipid-based mesophases in water. Some common examples include lamellar (L_α_), cubic (C), hexagonal (H), and inverted hexagonal phases (H_II_).^2−4^ In this phase space, the specific morphology adopted by a given lipid amphiphile is determined by a number of factors, including temperature, pressure, molecular structure and shape, membrane elasticity, and concentration.^5^

Unlike these water-insoluble lipids, many amphiphiles—such as detergents and soaps—are water-soluble. Below a threshold concentration, termed the critical micelle concentration (CMC), these surface-active agent molecules (or surfactants) coat interfacial surfaces and lower the surface tension, including gas, liquid, and solid interfaces.^6,7^ Above the CMC, surfactants organize into discrete spherical and cylindrical micellar mesostructures, which disperse in the bulk aqueous environment as a colloidal solution.^8−12^ The interactions between these micelle-forming surfactants and the equilibrated mesophases of insoluble lipids have been a subject of long-standing interest.^6^ This is because these interactions form the basis of many important technologies for the extraction, purification, crystallization, and reconstitution of membrane proteins, one of the most important classes of biomolecules targeted by prescription drugs.^13−15^

A significant body of previous research has led to a generalized model of surfactant–membrane interactions. En route to dissolution, a series of complex and reversible phase transformations from lipidic lamellar organizations to lipid-saturated mixed micelles to detergent-saturated mixed micelles when excess detergent is present in bulk aqueous solutions occurs.^16^ This mechanism is termed the three-stage model, first proposed by Helenius and Simons in 1975.^16^ The model pairs the above morphological changes to three different stages of local thermodynamic equilibration.

However, the thermodynamic equilibrium picture above does not fully describe the conditions in which surfactants and membranes interact where kinetics considerations dominate.^17,18^ A significant body of experimental and computational research on membrane–surfactant interactions suggests a more complex picture, which drives the surfactant-induced solubilization of the lipidic lamellar mesophases.^19,20^ Particularly, Nomura et al. examined the dynamics of interactions between surfactants and giant unilamellar vesicles (GUVs) in real time, documenting a variety of kinetic pathways that characterize the dissolution dynamics. These pathways were dependent on the physical properties of the membrane and the partitioning behaviors of the surfactant used.^18^ Extending these studies to the dissolution of different morphologies, a class of lipidic multilamellar cylindrical mesophases termed myelin figures, we found further evidence for how surfactant partitioning can affect the morphological evolution and the ultimate dissolution of the lamellar phase.^21^ Taken together, these observations support the notion that a thorough understanding of how surfactants partition within a membrane’s bilayers and the consequential deformations of the lipidic lamellar phase is needed to achieve a more complete understanding of surfactant–membrane behavior.

**Figure 1:**
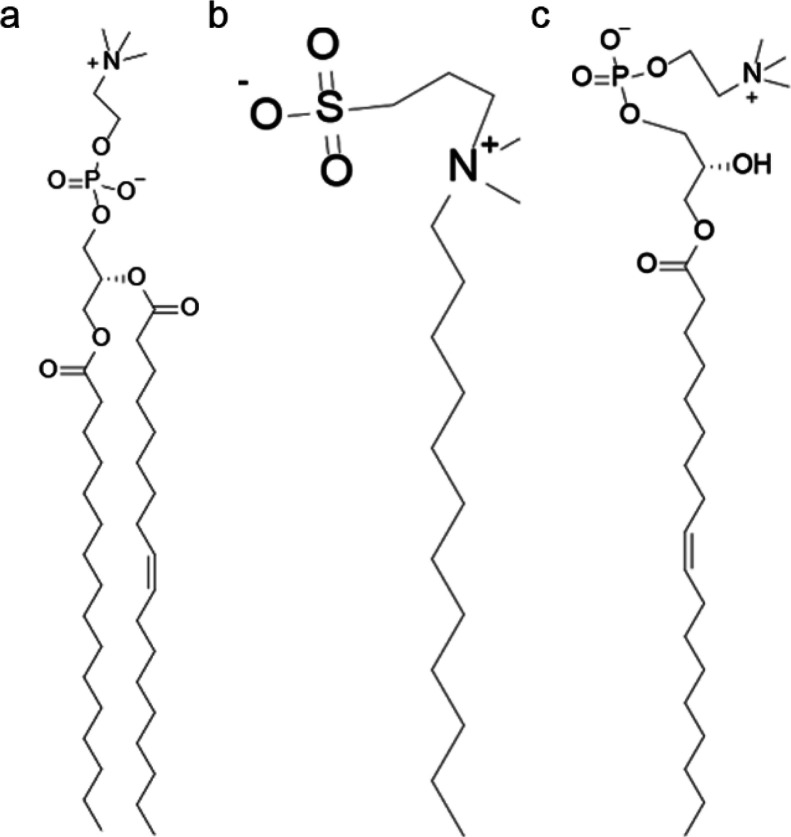
Chemical structure of the experimental amphiphiles POPC, DDAPS, and O-Lyso-PC. Chemical structures of (a) 1-palmitoyl-2-oleoyl-*sn*-glycero-3-phosphocholine (POPC), (b) *n*-dodecyl-*N*,*N*-dimethyl-3-ammonio-1-propanesulfonate (DDAPS), and (c) 1-oleoyl-2-hydroxy-*sn*-glycero-3-phosphocholine (O-Lyso-PC).

Here, we investigate the interactions and organization of the bilayer-forming water-insoluble phospholipid 1-palmitoyl-2-oleoyl-*sn*-glycero-3-phosphocholine (POPC) and the water-soluble, micelle-forming zwitterionic surfactant Zwittergent 3-12 (*n*-dodecyl-*N*,*N*-dimethyl-3-ammonio-1-propanesulfonate or DDAPS) (Figure 1). Concurrently, we studied the dynamics of another micelle-forming zwitterionic surfactant, 1-oleoyl-2-hydroxy-*sn*-glycero-3-phosphocholine (O-Lyso-PC) with the same procedures for comparison. In both cases, planar films of POPC to surfactant (POPC:surfactant) mixtures, between 100:1 and 1:4 molar ratios, deposited on solid supports are hydrated by water vapor in sealed humidity chambers containing saturated K_2_SO_4_ solutions (having a relative humidity, or RH, of 98%). The resulting morphologies are subsequently characterized using a combination of X-ray diffraction (XRD), brightfield optical microscopy, wide-field fluorescence microscopy, and atomic force microscopy (AFM).

Results presented here establish that the POPC:surfactant mixtures coassemble into well-ordered multilamellar mesophases for a wide range of molar ratios. While previous work shows that surfactants to form interfacial monolayers on solid supports, the assembly and preservation of the lamellar motif across the multilamellar stack with surfactant-dominated compositions is highly unique.^7,22^ Furthermore, we found that the partitioning of the surfactant within the lamellar lipid phase did not induce large-scale lipid–surfactant phase separation or distorted the lamellar phase to any noticeable degree. Instead, the two surfactants introduced subtle structural perturbations to the lamellar phase while preserving the multilamellar stack. With increasing DDAPS concentration, the lamellar spacing (*D*) of the POPC mesophases decreases monotonically. Decreases in *D* were driven by corresponding gradual decreases in both the headgroup-to-headgroup spacing (*D*_hh_) and the thickness of the interlamellar water layer (*D*_w_)—consistent with the surfactant-mediated “drying” and disordering of the hydrophobic space of the lamellar phase.^6^ By contrast, an increased concentration of O-Lyso-PC drives a surprising structural transition. Below a 1:2 molar ratio of POPC:O-Lyso-PC, the lamellar spacing of the POPC mesophases remains essentially unchanged. This apparent structural “stability” arises despite surfactant-induced disordering (as accompanied by a corresponding decrease in the headgroup-to-headgroup distance) of the lamellar phase. Curiously, the surfactant-induced disordering, which implies thinning, is counteracted and compensated for by a corresponding increase in the interlamellar water layer thickness. However, at a 1:2 molar ratio of POPC:O-Lyso-PC, both the water layer thickness and the headgroup distance decreases. Taken together, our results illustrate how the chemical complexities of surfactant–membrane interactions alter the structure of mixed mesophases and ultimately determine the kinetic pathways by which surfactants dissolve lamellar lipid mesophases.

## Materials and Methods

### Materials

1-Palmitoyl-2-oleoyl-*sn*-glycero-3-phosphocholine (POPC), 1,2-dioleoyl-*sn*-glycero-3-phosphoethanolamine-*N*-(lissamine rhodamine B sulfonyl) (ammonium salt) (Rho B-DOPE), and 1-oleoyl-2-hydroxy-*sn*-glycero-3-phosphocholine (O-Lyso-PC) were purchased from Avanti Polar Lipids (Alabaster, Al). *n*-Dodecyl-*N,N*-dimethyl-3-ammonio-1-propanesulfonate (DDAPS) was acquired from MilliporeSigma (Burlington, MA). Chloroform, methanol, and 2,2,2-trifluoroethanol were purchased from Sigma-Aldrich (St. Louis, MO). Silicon [100] wafers were acquired from Sigma-Aldrich (St. Louis, MO) and borosilicate microscope slides were obtained from Corning (Corning, NY). Nitrogen gas was acquired from Praxair (Danburt, CT). Deionized water was prepared with a Milli-Q Synthesis water purification system (>15 M-Ohm/cm; MilliporeSigma; Burlington, MA). All chemicals were used without further purification.

### X-ray Diffraction Sample Preparation, Measurements, and Analysis

XRD experiments were performed on multilamellar stacks of oriented lipid bilayers deposited on freshly cleaned hydrophilic silicon [100] wafers. Silicon substrates, cut to 18 × 20 mm, were sonicated for 15 min in methanol followed by another 15 min in deionized water a total of three times. Substrates were then nitrogen-dried and exposed to short-wavelength UV radiation for 30 min to make the surface hydrophilic.

The wafers were placed on an accurately leveled platform for amphiphile deposition. 0.002 mol of POPC and the desired amount of surfactant were dissolved in 200 μL of a 1:1 solution of chloroform:2,2,2-trifluoroethanol, and then the solution was deposited drop by drop on the silicon substrate. The wafer was left covered for 2 h in a fume hood for slow evaporation. It was then placed under high vacuum for 24 h to remove trapped solvents. The lipid-dried film was equilibrated under 98% relative humidity (RH) at a temperature of 50 °C for 48 h, and then, finally, it was equilibrated at room temperature for an additional 24 h at 98% RH, which was achieved by a reservoir filled with a saturated K_2_SO_4_ solution.^23^

The diffraction measurements were carried out using an in-house Cu Kα tube spectrometer with a wavelength of 1.54 Å operating in the horizontal plane. During the in-house X-ray diffraction measurements, we used a specially constructed humidity cell designed for high accuracy and sensitivity in RH.^24^ The scattered intensity was plotted as a function of *Q* (scattering vector), which is directly related to the scattering angle by *Q* = 4π sin(θ)/λ, where λ is the wavelength of the X-rays. Therefore, we obtained one-dimensional *I*(*Q*) profiles for each sample, showing up to nine Bragg orders of magnitude. The X-ray diffraction pattern presented a series of sequential peaks positioned at equal interpeak distances, characteristic of a lamellar phase. The diffraction peaks were fitted by Gaussians after background subtraction to determine their positions and areas under the peak. Miller indices (*hkl*) correspond to those of a lamellar phase for all studied samples: 001, 002, 003, .... The lamellar spacing (*D*) of the mesophase was calculated following Bragg’s law for a 1D crystal on a plot of peak location (*q*) vs diffraction order (*h*) and using the following equation: *D* = 2π/Δ*q*. The addition of surfactants leads to an expected higher disorder on the phospholipid lipid bilayers due to interference between the bilayer and surfactant molecules. However, for all diffraction patterns obtained, the full width at half-maximum (FWHM) of diffraction peaks remained between 0.005 and 0.007 for all samples, which indicates a similar quality in all the amphiphilic films. Moreover, XRD measurements show signatures of a form factor corresponding to possible thermal smectic fluctuations of lipid bilayers.^25,26^ However, an increase in peak widths has a minor effect compared to peak height changes.

The integrated intensity of *n*^th^ order peaks (*I*_*n*_) was then used to calculate the electron density profiles with the following equation:
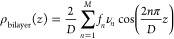
where the coefficients *f*_*n*_ can be found with the formula , *Q*_*z*_ is the Lorentz correction factor equal to *q* for oriented bilayers, and *ν*_*n*_ corresponds to the phase of the structure factor corresponding to the POPC.^27,28^ The phases used for each order were [−1, −1, +1, −1, +1, −1, −1, −1, −1]. Absorption correction for oriented samples was applied on intensities as described previously.^29^ Finally, the distance between the two characteristic maxima was attributed to the lipid headgroup to headgroup distance (*D*_hh_) along the bilayer normal, and the water layer thickness (*D*_w_) between lipid bilayers was defined as *D*_w_ = *D* – *D*_hh_.

### Lipid:Surfactant Sample Preparation for All Microscopy Techniques

Supported multilamellar membranes were prepared by adapting a similar method of liquid deposition and gaseous hydration to the section above.^28^ Borosilicate glass cover slides were cleaned by sonication in methanol and then deionized water for 15 min three times. The surface supports were then dried with nitrogen gas and treated by UV radiation (185 and 254 nm) for 30 min. Sample stock solutions were prepared by dissolving 1 μmol of POPC and 10 nmol of Rho B-DOPE in a 50% volume percent (v/v) solution of chloroform:2,2,2-trifluoroethanol. Then varying molar equivalents (in relation to POPC) of DDAPS or O-Lyso-PC were added and diluted to a final volume of 200 μL of the 1:1 v/v mixture. Sample stock solutions used for brightfield optical microscopy experimentation did not include Rho B-DOPE. Once cleaned, 50 μL of the prepared lipid stock solution was pipetted on to the surface supports on a level platform. The supports were covered by aluminum foil, and the solution was allowed to dry in the atmosphere for 2 h and then dried by house vacuum overnight. The surface supports were sealed in a humidity chamber and the relative humidity was elevated to 98% by a saturated K_2_SO_4_ solution for 24 h in a 55 °C oven. The surface supports were allowed to equilibrate to room temperature for at least 24 h at 98% RH. Care was taken to minimize errors caused by condensation within the seal (by careful handling of the chamber or transfer to another humidity chamber). Afterward, the surface supports were brought to the appropriate instrument for analysis or stored at room temperature in their sealed humidity chamber. All samples were used that same day or properly resealed for usage within 14 days.

### Brightfield Optical Microscopy Visualization

Brightfield optical microscopy measurements were performed using a Nikon Eclipse TE2000S inverted fluorescence microscope (Technical Instruments, Burlingame, CA) equipped with a Roper Cool Snap CCD camera (Technical Instruments, Burlingame, CA). Videos were taken by using a Plan Fluor 20X (NA, 0.25) air objective (Nikon, Japan). The resulting micrographs were processed by using the ImageJ software package.

### Wide-Field Fluorescence Microscopy and Image Analysis

Wide-field fluorescence microscopy measurements were performed using a Nikon Eclipse TE2000S inverted fluorescence microscope (Technical Instruments, Burlingame, CA) equipped with a Roper Cool Snap CCD camera (Technical Instruments, Burlingame, CA) and a Hg lamp as a light source. Videos were taken using a Plan Fluor 20X (NA, 0.25) air objective (Nikon, Japan) and filter cubes to filter the absorption and emission of the source and camera. All images and videos were collected with the samples still housed in the humidity chamber and analyzed by using the ImageJ software package. Fluorescence intensity was computed by measurements normalized to the maximum and background values of the surface supports.

### Atomic Force Microscopy Topography Investigation and Analysis

AFM images were acquired using a deflection type configuration (MFP-3D, Oxford Instrument, Santa Barbara, CA) following similar protocols reported previously.^30^ Silicon nitride probes (MSNL-10 E, *k* = 0.1 N/m, Bruker, Camarillo, CA) were used to characterize the topology of the printed structures. Image acquisition was done using tapping mode with 40–60% damping.^31,32^ Image processing and display were performed by using the MFP-3D software developed on the Igor Pro 6.20 platform. Supported multilamellar membranes were prepared following the same methods as those for wide-field fluorescence microscopy imaging.

The force versus distance profiles were acquired by approaching the probe to the lipid constructs from above at a constant velocity (100 nm/s). The vertical force applied to the amphiphilic mesophases was known to perturb the interactions between molecules.^33^ The spring constant of each probe was calibrated based on measurements of thermal fluctuations of the cantilever.^34^ All experiments were performed at 24 °C in a temperature-controlled room with stability of ±1 °C. Force–distance plots were displayed and analyzed using the MFP-3D software developed on the Igor Pro 6.20 platform.

## Results and Discussion

We begin by characterizing the lamellar mesophase consisting of just the single phospholipid, POPC, at room temperature by using XRD. A detailed analysis of the data obtained (see “Materials and Methods” section above) yielded the values for the three lamellar periodicities: a lamellar spacing (*D*) of 51.8 Å, a headgroup-to-headgroup distance (*D*_hh_) of 39.4 Å, and a water layer thickness (*D*_w_) of 12.4 Å. It is important to note that *D* includes the water layer between the lipid bilayers. These values are in statistical agreements with those reported previously.^35−38^

To enable visualization of the lamellar mesophase by wide-field fluorescence microscopy, we doped a POPC stock solution with 1 mol % Rho B-DOPE. Visualizing the lamellar mesophase prepared from this doped solution revealed a homogeneous fluorescence intensity after normalization to the background and maximum value (94 ± 2.7% of max fluorescence intensity) across a line plot on the surface, consistent with a uniform, lamellar organization (Figure S1).

**Figure 2:**
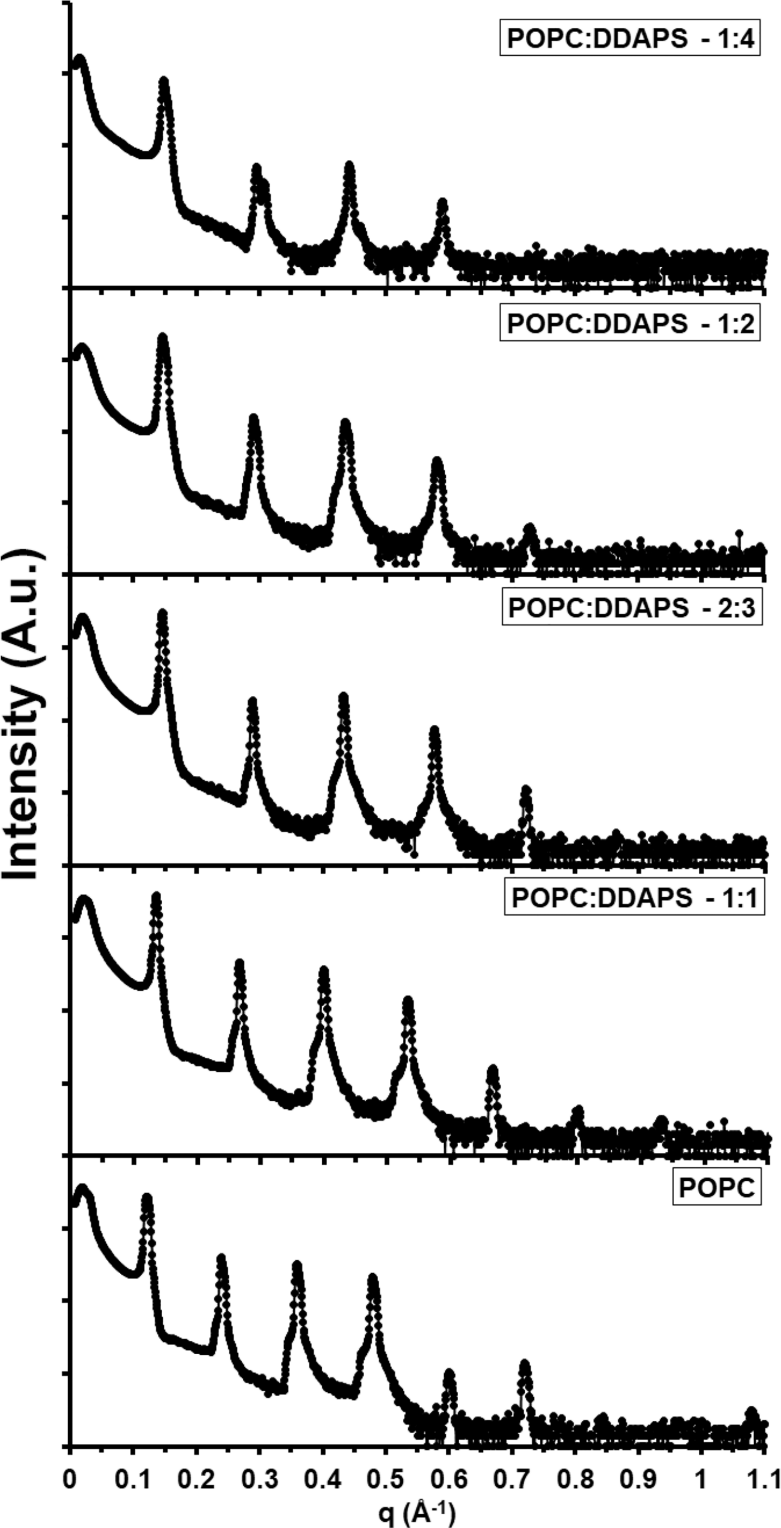
Experimental XRD data of POPC:DDAPS mesophases. A stacked plot of the intensities of the X-ray diffraction peaks of various POPC:DDAPS multilamellar mesophases was constructed. The molar ratios plotted include 1:0, 1:1, 2:3, 1:2, and 1:4 POPC:DDAPS.

**Figure 3:**
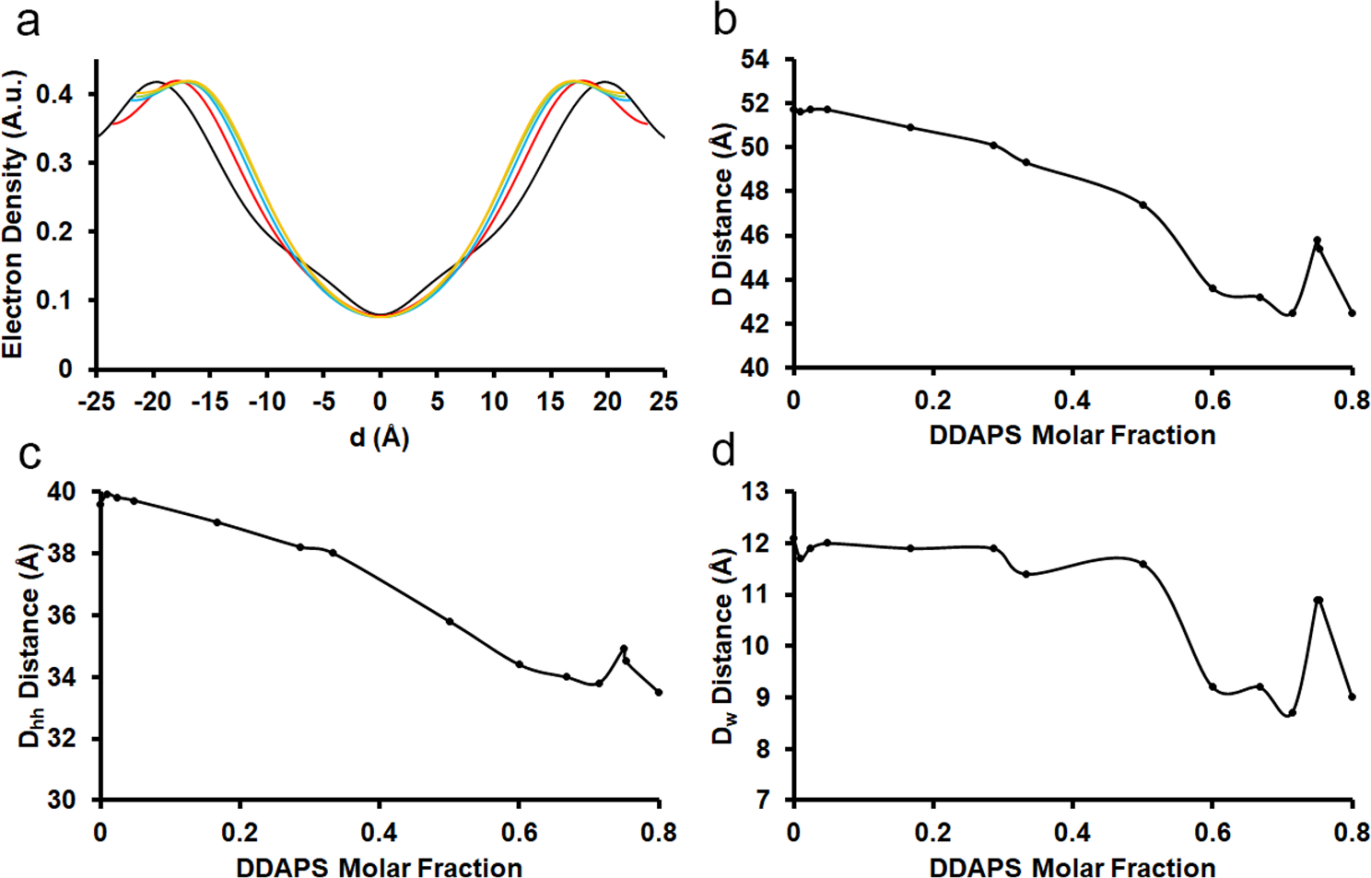
Lamellar structure of POPC:DDAPS multilamellar mesophases by molar fraction. (a) Average electron densities normal to the bilayers were assembled from the diffraction peaks and plotted with an arbitrary scale. The molar ratios plotted include 1:0 (black), 1:1 (red), 2:3 (blue), 1:2 (green), and 1:4 (orange) POPC:DDAPS. (b) Lamellar spacing (*D*) of POPC:DDAPS multilamellar mesophases was deduced by XRD and plotted by the molar fraction of DDAPS. (c) The headgroup-to-headgroup distances (*D*_hh_) of POPC:DDAPS multilamellar mesophases were calculated by XRD and plotted by molar fraction of DDAPS. (d) Water layer thickness (*D*_w_) was calculated from *D* and *D*_hh_ and was similarly plotted.

Next, we examined lamellar mesophases produced from mixtures of POPC and the surfactant DDAPS with systematically varied lipid:surfactant molar ratios (100:1, 40:1, 20:1, 5:1, 5:2, 2:1, 1:1, 2:3, 1:2, 2:5, 1:3, 20:61, and 1:4) using XRD measurements. *First*, we found that the lamellar motif was remarkably preserved across a broad concentration range. This is evident in the existence of single, well-defined lamellar repeat distances found in the XRD measurements (Figure 2 and Figure S2). The preservation of the lamellar order in the mixed mesophase is particularly surprising since there is a significant mismatch in the spontaneous curvatures between DDAPS (presumedly *J* ≫ 0 Å^–1^) and POPC (*J* = −0.0022 ± 0.0010 Å^–1^).^21,39^ The disparity should be sufficient to drive the surfactant to phase segregate and deform the lamellar organization. At present, we do not understand the robust preservation of the lamellar order. However, the lipid–surfactant system easily dissolves when adding excess bulk water (Figure S3 and Videos S1–S9), therefore suggesting that our experimentally low amounts of water in the system could correlate with lamellar phase assembly within a larger phase diagram.^40,41^*Second*, the addition of DDAPS molecules to the POPC bilayer stacks showed variable patterns of structural modulation (Figure 3). Up to a 2:3 molar ratio of POPC:DDAPS, lamellar spacing decreased gradually to a value of 43.6 Å. Concurrently, headgroup distance decreased to a near minimum of 34.4 Å at the same concentration. Above this concentration to a 1:4 molar ratio, *D* and *D*_hh_ marginally thinned to 42.5 and 33.5 Å, respectively. In contrast, water layer thickness exhibited nonlinear trends over an increasing concentration of DDAPS. At first, *D*_w_ hovered between 12.1 to 11.4 Å up to a 1:1 molar ratio but declined to 9.2 Å at a 2:3 molar ratio. Increasing the DDAPS concentration beyond a 2:3 molar ratio minimally impacted the water layer thickness except for an errant value of 10.9 Å around a 1:3 molar ratio. *Third*, visualizing the lipid–surfactant mixed mesophase by wide-field fluorescence microscopy displayed no significant morphological disruptions (Figure S4). Selected POPC:DDAPS mixtures (1:1, 1:2, 1:3, and 1:4) were doped with 1 mol % Rho B-DOPE of the POPC concentration, and lamellar mesophases were promptly assembled. Normalized fluorescence intensity values were examined on a line plot across the surface, and statistically homogeneous intensities (94 ± 2.6%, 91 ± 4.4%, 92 ± 2.9%, and 91 ± 3.4%, for 1:1, 1:2, 1:3, and 1:4 molar ratios, respectively) were observed. These observations further confirm the lamellarity of this mesophase and demonstrate a lack of significant perturbations. However, at the highest DDAPS molar fractions (≥a 2:5 molar ratio) XRD data showed a small but detectable peak splitting toward higher *q*-values indicating a loss of total sample homogeneity. We anticipate that this could be a consequence of excess DDAPS within the mesophase.

**Figure 4:**
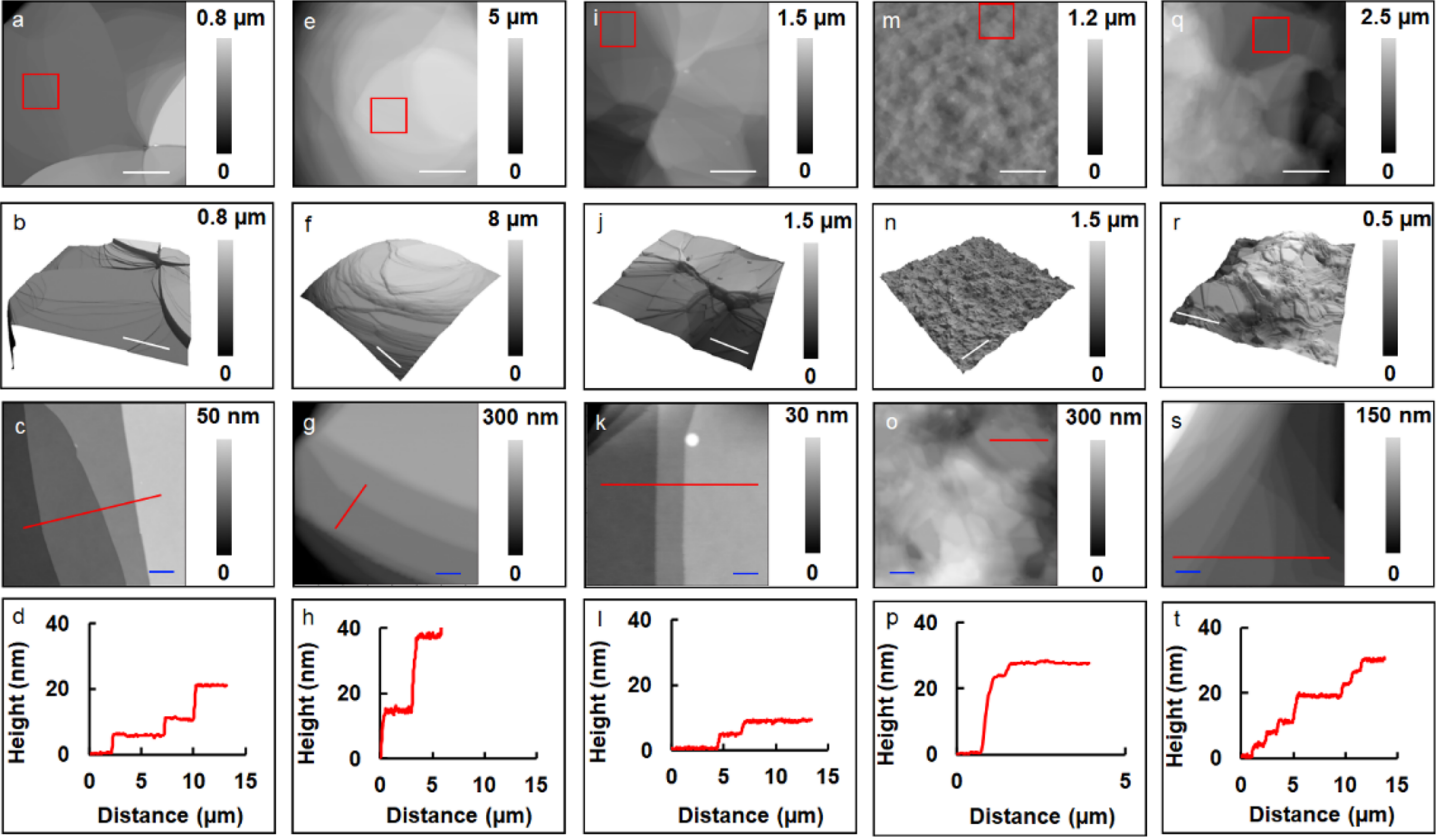
AFM topographic images of POPC:DDAPS mesostructures. (a) AFM topographic image of 1:0 molar ratio POPC:DDAPS. (b) A 3D display of (a). (c) The AFM topographic image of the area indicated by the red square in (a). (d) A cursor profile of height over the mesophase’s surface as indicated by the red line in (c). (e) An AFM topographic image of 1:1 molar ratio POPC:DDAPS. (f) A 3D display of (e). (g) The AFM topographic image of the area indicated by the red square in (e). (h) A cursor profile of height over the mesophase’s surface as indicated by the red line in (g). (i) An AFM topographic image of 1:2 molar ratio POPC:DDAPS. (j) A 3D display of (i). (k) The AFM topographic image of the area indicated by the red square in (i). (l) A cursor profile of height over the mesophase’s surface as indicated by the red line in (k). (m) An AFM topographic image of 1:3 molar ratio POPC:DDAPS. (n) A 3D display of (m). (o) The AFM topographic image of the area indicated by the red square in (m). (p) A cursor profile of height over the mesophase’s surface as indicated by the red line in (o). (q) An AFM topographic image of 1:4 molar ratio POPC:DDAPS. (r) A 3D display of (q). (s) The AFM topographic image of the area indicated by the red square in (q). (t) A cursor profile of height over the mesophase’s surface as indicated by the red line in (s). Blue scale bar = 2 μm, white scale bar = 20 μm.

**Figure 5:**
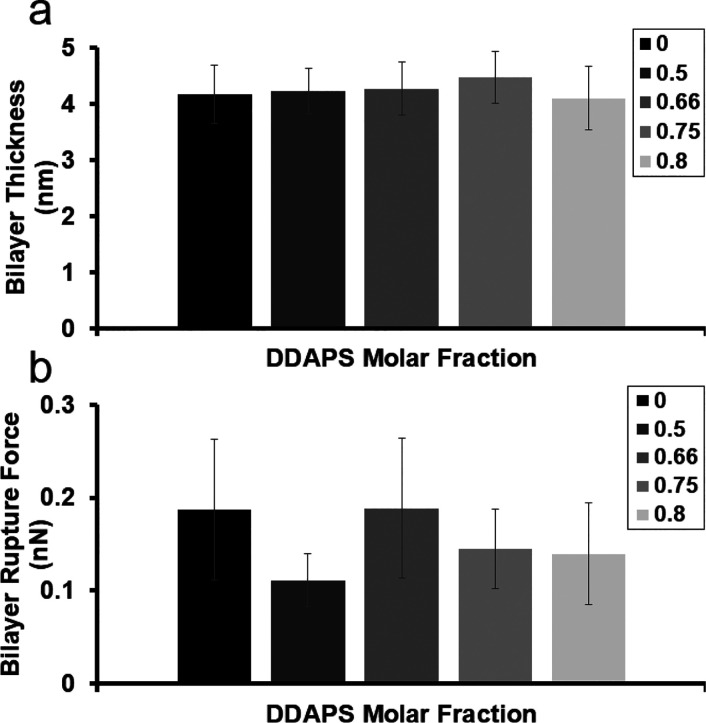
Bilayer thickness and rupture force of POPC:DDAPS multilamellar mesophases by molar fraction as measured by AFM. (a) Bilayer thickness (*D*_t_) of POPC:DDAPS multilamellar mesophases was deduced by AFM across the entire stack, averaged, and plotted by molar fraction of DDAPS. Error bars are standard deviations. (b) Bilayer rupture force (*F*_r_) of POPC:DDAPS multilamellar mesophases was similarly elucidated and plotted by molar fraction of DDAPS. Error bars are standard deviations.

To further understand the structural properties of these mixed multilamellar mesophases, samples were assembled from selected mixtures of POPC:DDAPS with 1 mol % Rho B-DOPE as a dopant and investigated utilizing AFM measurements. First, AFM topographic images were acquired for the systems consisting of 1:0, 1:1, 1:2, 1:3, and 1:4 molar ratios of POPC:DDAPS, respectively, all of which contained 1 mol % Rho B-DOPE dopant (Figure 4). Topographic images clearly show plateaus and steps present among the 1:0, 1:1, and 1:2 samples (Figure 4f). At the edges of these materials, the step height of the stacked lamellae were measured to be consistent with the integer multiples of POPC bilayers (4.51 nm).^42^ Therefore, these mesostructures are likely bilayer stacks similar to those prepared using the drop-and-dry method. At higher concentration ratios of DDAPS, the terraced steps are less smooth than samples with lower ratios of DDAPS (Figure 4n,r). Such structural dissonance could be a consequence of the unincorporated DDAPS as mentioned above. A commensurate edge of a different multilamellar stack with a 1:2 molar ratio of POPC:DDAPS and 1 mol % Rho B -DOPE was visualized using wide-field fluorescence microscopy, exhibiting a nonquantized increase in fluorescence intensity across the terrace morphology (Figure S5). Such an observation highlights the increased capability of AFM as a high-resolution technique for such structural analysis in future studies. Next, we measured the surface force curves (see “Materials and Methods” section above) of several selected mixtures of POPC:DDAPS (1:0, 1:1, 1:2, 1:3, and 1:4) with 1 mol % Rho B-DOPE. From these examinations, two properties could be determined: the bilayer thickness (*D*_t_) and the bilayer rupture force (*F*_r_) (Figure 5 and Figure S6).^30^ Surprisingly, *D*_t_ and *F*_r_ displayed no significant change in value (∼4.2 nm and ∼0.15 nN, respectively) or correlation to surfactant concentration. Notably, the *F*_r_ values are an order of magnitude smaller than previously determined values of other liquid-crystalline bilayer mesophases.^30^ We anticipate that the surfactant-induced packing disruption and nontrivial lyotropic arrangements of the hydration network in these mesophases highly modulate lamellar mechanical properties.^43,44^ It is also worth noting that discrepancies between measurements of *D*_t_ and *D* originate from the instrumental techniques as AFM provides precise measurement on local membranes nanomechanical properties, whereas XRD provides information about the global average modulations in lamellar structures.^30,33^

**Figure 6:**
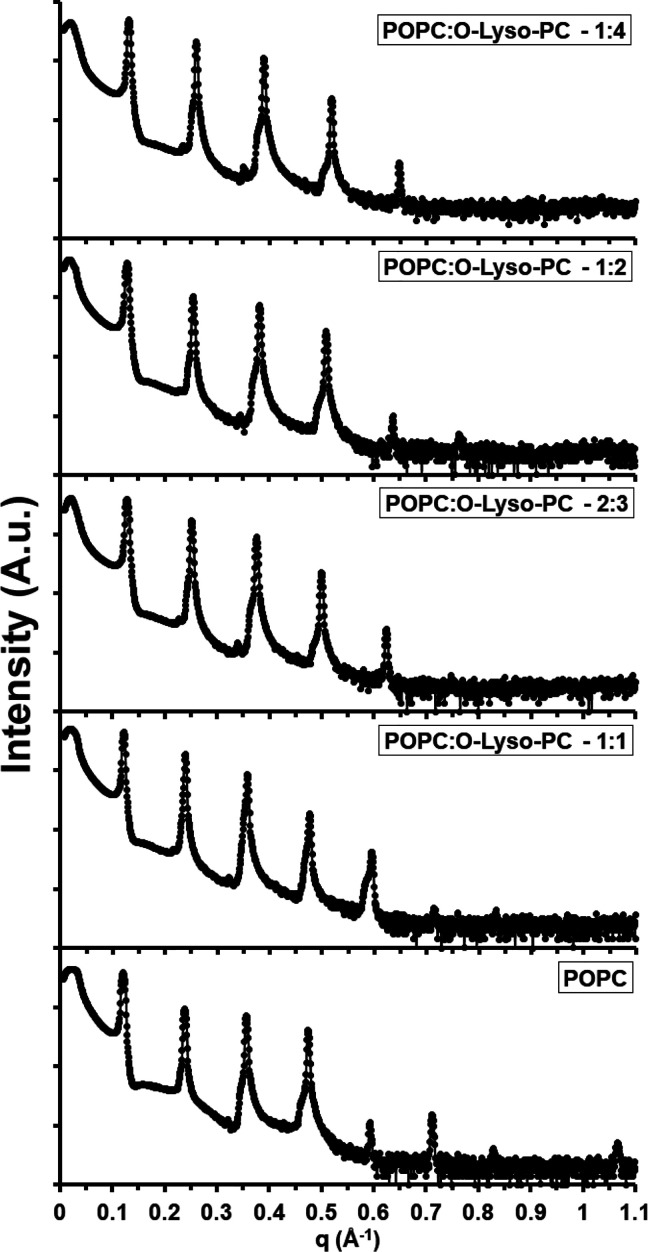
Experimental XRD data of POPC:O-Lyso-PC mesophases. A stacked plot of the intensities of the X-ray diffraction peaks of various POPC:O-Lyso-PC multilamellar mesophases was constructed. The molar ratios plotted include 1:0, 1:1, 2:3, 1:2, and 1:4 POPC:O-Lyso-PC.

**Figure 7:**
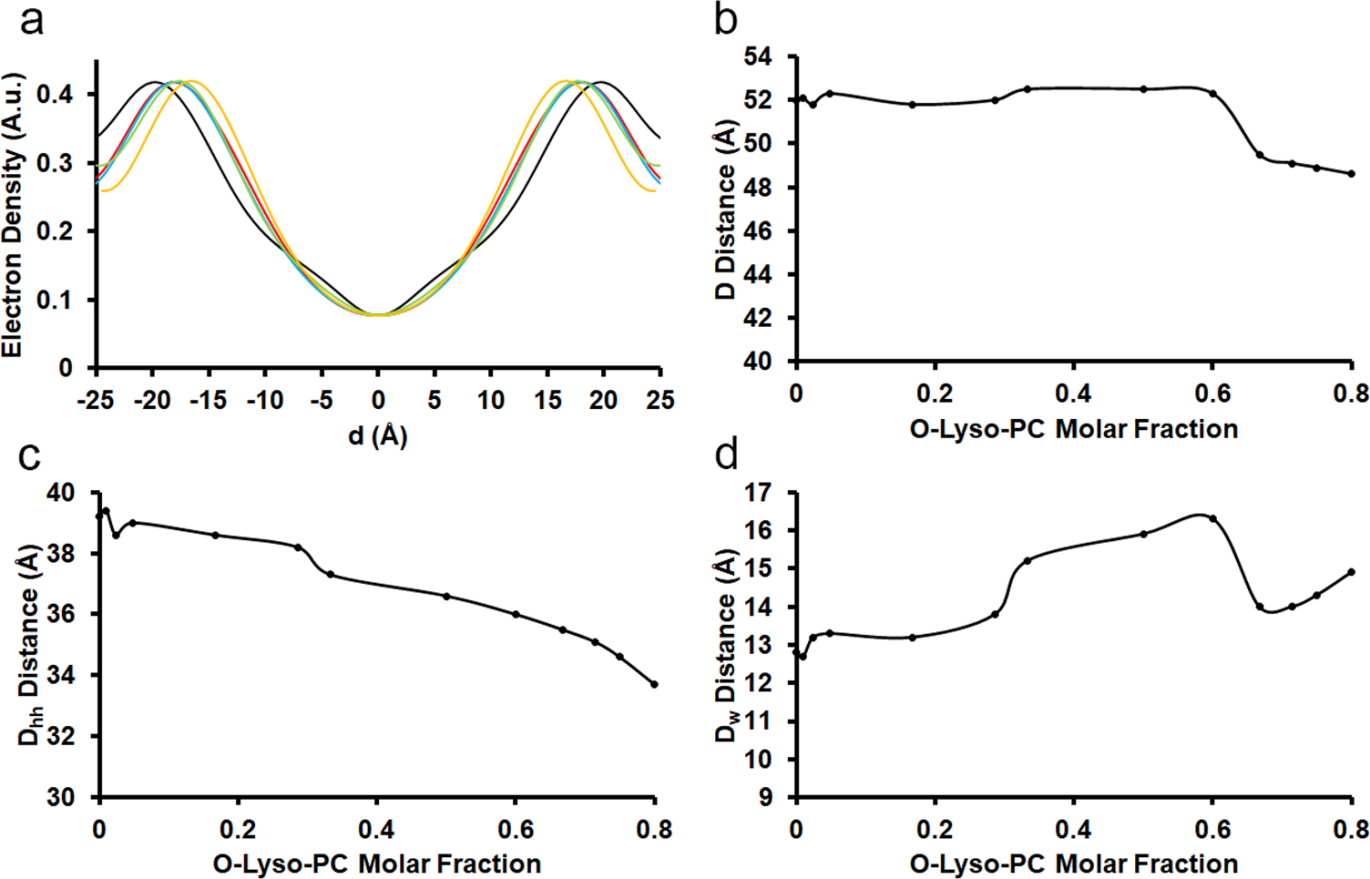
Lamellar structure of POPC:O-Lyso-PC multilamellar mesophases by the molar fraction. (a) Average electron densities normal to the bilayers were assembled from the diffraction peaks and plotted with an arbitrary scale. The molar ratios plotted include 1:0 (black), 1:1 (red), 2:3 (blue), 1:2 (green), and 1:4 (orange) POPC:DDAPS. (b) Lamellar spacing (*D*) of POPC:O-Lyso-PC multilamellar mesophases were deduced by XRD and plotted by molar fraction of O-Lyso-PC. (c) The headgroup-to-headgroup distance (*D*_hh_) of POPC:O-Lyso-PC multilamellar mesophases was calculated by XRD and plotted by molar fraction of O-Lyso-PC. (d) Water layer thickness (*D*_w_) was calculated from *D* and *D*_hh_ and similarly plotted.

Considering these findings, the dynamics of another micelle-forming zwitterionic surfactant, O-Lyso-PC (*J* = 0.0263 Å^–1^), was investigated when mixed with POPC in the lamellar mesophases (at molar ratios of 100:1, 40:1, 20:1, 5:1, 5:2, 2:1, 1:1, 2:3, 1:2, 2:5, 1:3, and 1:4).^39^ From a chemical perspective, the morphology of the O-Lyso-PC differs from the morphology of the DDAPS in multiple properties: CMC (being 2–4 mM for DDAPS and ∼10^–7^–10^–6^ M for O-Lyso-PC), headgroup structure (a sulfobetaine group for DDAPS and a phosphocholine group for O-Lyso-PC), and hydrophobic chain tail length (12 carbon atoms for DDAPS and 18 carbon atoms with a double bond at the ninth position for O-Lyso-PC).^45^ Modeling these two molecules have also displayed a difference in their headgroup hydration (in the form of their hydrogen bond acceptor count), being 3 for DDAPS and 7 for O-Lyso-PC in comparison to 8 for POPC.^46−48^ The resulting XRD measurements elucidated trends in the structural properties for this mixed lipid–surfactant system. *First*, the lamellar motif of these mixed mesophases was maintained across the entire concentration range of the O-Lyso-PC (Figure 6). Again, these findings are notable due to the large discrepancy in curvature between the involved amphiphiles. *Second*, the addition of O-Lyso-PC perturbed the structural properties of the lamellar motif with a unique directionality (Figure 7). Lamellar spacing (*D*) stayed mildly constant around a value of 52.1 Å from a 100:1 to a 2:3 molar ratio of POPC:O-Lyso-PC. At a 1:2 molar ratio, it markedly decreased to a value of 49.5 Å. With larger amounts of O-Lyso-PC, *D* monotonically decreased to a value of 48.6 Å at a 1:4 molar ratio. However, *D*_hh_ decreased continuously across the range of concentrations of O-Lyso-PC, beginning at 39.2 Å and ending at 33.7 Å for 100:1 and 1:4 molar ratios, respectively. Therefore, the nonlinear behavior of the trend in *D* can mainly be attributed to the variance in *D*_w_. Up to a 2:3 molar ratio, the water layer thickness surprisingly increased from 12.8 to 16.3 Å. At a 1:2 ratio of POPC:O-Lyso-PC, the water layer thickness pointedly decreased to 14.0 Å. Beyond this concentration of the O-Lyso-PC, the thickness marginally increased to 14.9 Å at a molar ratio of 1:4. *Third*, no morphological anomalies were observed upon visualization by wide-field fluorescence microscopy (Figure S7). Certain POPC:O-Lyso-PC mixtures (1:1, 1:2, 1:3, and 1:4 molar ratios) were doped with 1 mol % Rho B-DOPE of the POPC concentration, and lamellar mesophases were similarly assembled. Normalized fluorescence intensity values were examined on a line plot across the surface, and a statistically homogeneous intensity (95 ± 3.2%, 84 ± 6.3%, 93 ± 2.8%, and 94 ± 2.1%, for 1:1, 1:2, 1:3, and 1:4 molar ratios, respectively) was observed. These observations foreground the homogeneity of the lamellar mesophases without perturbations by physical phenomena, unlike the previous surfactant-rich POPC:DDAPS mesophases.

Given the results presented above, we investigated the necessity of POPC to form the lamellar motif within these mixed mesophases. An identical procedure of mesophase assembly was followed with a 0:1 molar ratio of POPC:DDAPS and POPC:O-Lyso-PC with Rho B-DOPE doped at a 1 mol % of the surfactant concentration. Visualizing the resulting morphologies with wide-field fluorescence microscopy featured little to no multilamellar mesophases (Figure S8). The amphiphilic mixtures, after water vapor hydration, appeared as amorphous “islands” with no indication of multilamellar stacks. Such results indicate that POPC is necessary for the formation of the lamellar motif. Further analysis can describe the concentration-dependent relationship of the phase behavior of this ternary system.

The assembly of POPC:DDAPS and POPC:O-Lyso-PC multilamellar mesophases by water vapor hydration and their varied trends in structural properties elicit numerous questions. Primarily, how is the solubilizing action of detergents inhibited at ratios of POPC to surfactant greater than the saturation limit of POPC bilayers?^16,49,50^ And, where do the different trends in structural properties originate as surfactant concentrations change? We believe that the proceeding insights foreground the need to abandon the three-stage model and create inclusive models of membrane-detergent mechanistic action..^18^

We begin by examining our experimental medium, water. Water has displayed an acute level of complexity as a solvent and a local environmental medium. For example, terahertz time-domain spectroscopy found that the hydration network surrounding a self-assembling amphiphilic polymer differentiated depending on the phase of its assembled mesophase.^51^ Specifically, the adjacent two layers of water molecules modulated in tune with amphiphile self-assembly. Further, past efforts have found a differentiated hydration network structure between the inside and outside of a multilamellar cylindrical assembly of cardanyl glucosides.^52^ Another example of this principle occurs when exchanging the sodium from sodium didodecyl sulfosuccinate to lithium, aqueous solubility and lateral headgroup area dramatically increases due to changes in its resulting hydration network.^53^ Other such works exhibit similar findings: mixed zwitterionic-anionic micellar mesophases modulate the CMC and precipitation phase boundaries (and, therefore, the surrounding hydration network). This is in contrast to the pure mesophases of the anionic amphiphile structures.^54^ An array of studies exhibits a mutualistic relationship between environment and an experimental system’s assembly.^55−57^ Therefore, our results, displaying surfactant-specific trends in the hydration network, agree with the notion that the chemical composition of the experimental mesophase modulates the hydrating water network.

**Figure 8:**
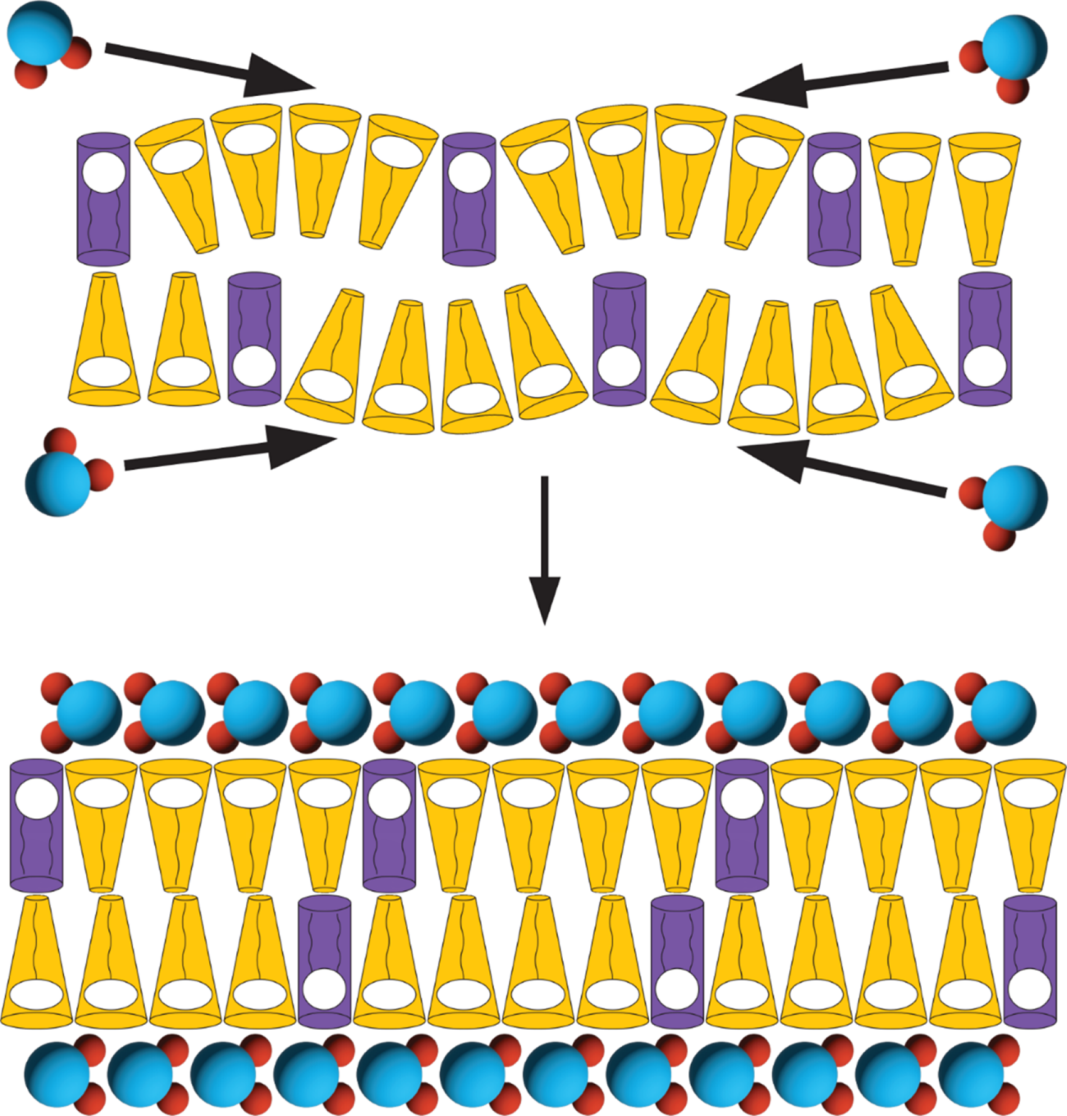
Water vapor-mediated assembly of multilamellar mesophases. A cartoon representation of a proposed mechanism of the water vapor hydration of the POPC:surfactant mixtures into multilamellar mesophases. Dried mixtures of POPC (purple cylinders) and surfactants (yellow cones) are hydrated by the surrounding water (blue and red models) within the humidity chamber, and lyotropic networks of amphiphiles and water assemble. In this mechanism, the morphological consequences of differentiated spontaneous curvature (and therefore solubilization) are repressed upon hydration due to energetic considerations of bilayer bending and the hydration network reorganizing.

Next, we focus on the nontrivial properties of lipidic multilamellar mesophases. This lyotropic arrangement of lamellae merits itself as an interesting subject of study due to its prevalence in nature, including plant chloroplasts and lamellar bodies.^58−60^ The organization of these smectic layers is stabilized by interlamellar interactions (or Helfrich interactions) which separates individual bilayers.^61^ Between the bilayers, an interstitial water layer exists which hydrates the amphiphiles’ headgroups allowing for the exchange of monomers between layers.^61^ Structurally locked by this balance of van der Waals forces and the hydrating network, participating amphiphiles still possess lateral fluidity among their neighbors.^62−64^ One area of interest about these mesophases is what happens when an adjacent bilayer is perturbed? Recent efforts by our group have exhibited the ability of these stacks to couple their behaviors three-dimensionally during events such as domain-forming phase separation.^28^ From this, it was proposed that the dynamics of headgroup hydration encourage the interlayer alignment of phases across the membranous stack. Peculiar hydration dynamics are not limited to just planar stacks but are also found in cylindrical multilamellar tubes (termed myelin figures) as well.^65^ Such complexities in amphiphile behavior can be exploited for morphogenesis, especially in response to external dopants like the detergents of focus in this work.^21^ However, here the involved surfactants are distributed within the amphiphilic mixture prewater vapor assembly. Therefore, these detergents should be randomly arranged within the multilamellar mesophase and not doped externally. Given this knowledge, we suggest that the solubilizing activity of the focal detergents is inhibited within multilamellar frameworks by the energetic cost of morphologically bending the membranes. This is compounded by the energetic cost to reorganize the lyotropic correspondence of adjacent bilayers and hydration networks within the stack (Figure 8).^5^

To study the impact of the surfactant’s chemical properties on the lamellar stack, we investigated the amphiphile’s headgroup. In our work, both of the participating surfactants have zwitterionic headgroups at the pH of deionized water. Alone, this leads to a large discrepancy in their ability to intercalate within the bilayer and flip-flop into the other leaflet.^66,67^ Since these amphiphiles translocate so slowly, the surfactants will presumably accumulate locally in their resident leaflet instead of equilibrating across the bilayer.^6^ Because of this, both the spatial configuration of the charges on the atomic components in the headgroup and their local population within the lamellae could nontrivially impact structural considerations. As an example, investigations of micellized sulfobetaine headgroups, like that in DDAPS, determined that ion spatial arrangement is influenced by the minimization of dipole–dipole repulsion, local entropic costs, and the maximization of hydration.^68,69^ Significantly, there is a calculated discrepancy in the hydrogen bond acceptor count of the two surfactant species and POPC (as computed by Cactvs 3.4.8.18 and released by PubChem), being 3 for DDAPS, 7 for O-Lyso-PC, and 8 for POPC.^46−48^ Such discrepancies in hydration can influence the packing of lipid-lysolipid mixed mesophases.^70^ These competing thermodynamic properties are further complicated by the variance of intermolecular interaction energies with bilayer composition: amphiphiles with identical chain lengths and different headgroups doped into lipidic lamellae can maximize their intermolecular interaction energies at different molar ratios. This foregrounds the consequences of differential van der Waals and electrostatic forces in structural behavior once intercalated into the bilayer.^71^ Therefore, it is reasonable to suggest that the arrangement of the phosphocholine and sulfobetaine headgroups will modulate structural and hydration behavior relative to POPC:O-Lyso-PC mesophases.^72^

Second, we consider the hydrophobic chain tails. In our work, our experimental detergents have different tails: a C12:0 alkyl chain for DDAPS and a C18:1 (Δ^9^) acyl tail for O-Lyso-PC, in comparison to POPC with a C18:1 (Δ^9^) acyl tail and a C16:0 acyl tail. It is important to note that a significant array of experimental works have elicited a generalized surfactant-induced disordering of the nonpolar space populated by the hydrophobic chain tails within bilayers.^6^ The mismatch of van der Waals interactions between the lipid’s and surfactant’s hydrophobic components perturbs the bilayer’s packing, leading to its thinning.^6,73^ Such perturbation can explain previously accounted behavior within multilamellar myelins, surfactant-mediated bilayer thinning can affect the exterior bilayers and initiate the twisting event of the multilamellar system.^21^ Our work highly aligns with this principle, as seen between the strong negative correlation between surfactant concentration and headgroup-to-headgroup distance measurements. However, it is worth noting that our regressions of *D*_hh_ with the surfactant concentration are component specific. We propose that this minute variability is a consequence of changes in van der Waals interactions due to the different chemical structures, consistent with previous literature. For example, lysolipid detergents can increase fluidity and decrease the bending rigidity of phospholipid membranes, exhibiting a positive correlation between chain length and bending rigidity.^74,75^ Furthermore, the same investigation found that the partitioning coefficients of the same lysolipid detergents within membranes correspond with acyl chain length.^75^ This is similarly true for amphiphiles with sulfobetaine headgroups doped into lipidic bilayers, longer hydrophobic chain tails correlate with stronger partitioning and a higher potential to micellize.^76,77^ Further investigation found that hydrophobic chain tails of DDAPS can fold back on themselves within micellar structures, foregrounding their potential for packing mismatch.^78^ Such results align with our proposal above, signifying that molecule-specific hydrophobic chain tail mismatch likely determines the value of *D*_hh_ and its relative regressions.

## Conclusion

Here, multilamellar mesophases composed of POPC, POPC:DDAPS, and POPC:O-Lyso-PC mixtures were assembled by water vapor hydration and investigated with XRD techniques. Such mixed mesophases were formed across the entire concentration range employed, between 100:1 and 1:4 molar ratios of POPC:surfactant. Notedly, POPC:surfactant mixtures dissolved as an isotropic solution of mixed micelles when hydrated by bulk liquid water instead as visualized by brightfield optical microscopy. Lamellar spacing, headgroup-to-headgroup distance, and water layer thickness of the assembled bilayers were all calculated from the XRD measurements. Generally, *D* decreased with larger amounts of surfactant and *D*_hh_ decreased monotonically with increasing surfactant concentration. However, the trends of *D*_w_ were highly variable with surfactant incorporation. *D*_w_ of POPC:DDAPS mesophases stayed mildly constant (around 11.8 Å) until a decline to 9.2 Å at a 2:3 molar ratio. In contrast, POPC:O-Lyso-PC mesophases displayed an increase of *D*_w_ from 12.8 Å at a 100:1 molar ratio to 16.3 Å at a 2:3 molar ratio, which proceeded by a decrease to 14.0 Å at a 1:2 molar ratio. Selected molar ratios (1:1, 1:2, 1:3, and 1:4) of POPC:DDAPS and POPC:O-Lyso-PC mixtures were doped with 1 mol % Rho B-DOPE of the POPC concentration, assembled into multilamellar stacks, and visualized by wide-field fluorescence microscopy. This resulted in negligent perturbation of the lamellar motif by physical phenomena like phase separation events. Identical mixtures of POPC:DDAPS with 1 mol % Rho B-DOPE dopant were assembled into multilamellar mesophases and investigated using AFM. Such measurements indicated a homogeneous topography across the multilamellar stack for surfactant-poor and surfactant-rich samples with bilayer thickness (*D*_t_) bilayer rupture force (*F*_r_) hovering around ∼4.2 and 0.15 nN, respectively, for the entire surfactant concentration range.

Our findings regarding these unique multilamellar mesophases suggest a wide scope of conclusions. *First*, these mesophases, composed of a wide range of POPC:surfactant molar ratios, foreground an interesting water-deficient phase behavior region. Further research could describe a complete depiction of the phase diagrams (POPC:DDAPS/O-Lyso-PC:water), leading to a more holistic understanding of our surfactant–membrane systems. *Second*, the variance in trends of structural properties highlights the consequential, nontrivial thermodynamic interactions of our experimental components like the chemical potential of the hydrating water, the van der Waals interactions of the chemical structures, and the electrostatic considerations during packing. Specifically, the hydration of the hydrophilic headgroups and the mismatch of the hydrophobic chain tails likely dictate the structural properties of the involved bilayers. Such considerations are not traditionally considered when examining the macroscale morphological changes of surfactant-membrane systems.^18^*Third*, this work agrees with the sentiment that water is not just a bulk solvent but an involved component of this self-assembling system. Such a shift in the experimental framework can inform future scientific efforts in self-assembly and possibly lead to the development of novel solubilization assays for the efficient sequestering of membrane-bound proteins using solid-adsorbed surfactant material. Taken together, we stress that our understanding of surfactant activity must move beyond the three-stage model and into a kinetically and chemically complex world.

## Supporting Information Available

Hydration of dried POPC by direct contact with liquid water (AVI)
Hydration of dried 1:1 POPC:DDAPS by direct contact with liquid water (AVI)
Hydration of dried 1:2 POPC:DDAPS by direct contact with liquid water (AVI)
Hydration of dried 1:3 POPC:DDAPS by direct contact with liquid water (AVI)
Hydration of dried 1:4 POPC:DDAPS by direct contact with liquid water (AVI)
Hydration of dried 1:1 POPC:O-Lyso-PC by direct contact with liquid water (AVI)
Hydration of dried 1:2 POPC:O-Lyso-PC by direct contact with liquid water (AVI)
Hydration of dried 1:3 POPC:O-Lyso-PC by direct contact with liquid water (AVI)
Hydration of dried 1:4 POPC:O-Lyso-PC by direct contact with liquid water (AVI)
Figures showing POPC multilamellar stack fluorescence intensity, indexed *Q*-values of POPC and POPC:DDAPS lamellar mesophases, surface hydration of dried POPC:surfactant mixtures, POPC:DDAPS multilamellar stack fluorescence intensity, POPC:DDAPS multilamellar stack edge fluorescence intensity, indexed *Q*-values of POPC and POPC:O-Lyso-PC lamellar mesophases, POPC:O-Lyso-pc multilamellar stack fluorescence intensity, DDAPS and O-Lyso-PC assemblies upon water vapor hydration (PDF)

## Supplementary Material

jp3c01654_si_001.avi

jp3c01654_si_002.avi

jp3c01654_si_003.avi

jp3c01654_si_004.avi

jp3c01654_si_005.avi

jp3c01654_si_006.avi

jp3c01654_si_007.avi

jp3c01654_si_008.avi

jp3c01654_si_009.avi

## ABBREVIATIONS

AFM: atomic force microscopy
C: cubic phase
CHOL: cholesterol
CMC: critical micelle concentration
D: lamellar spacing
*D*_hh_: headgroup-to-headgroup distance
*D*_t_: bilayer thickness
*D*_w_: water layer thickness
DDAPS: *n*-dodecyl-*N*,*N*-dimethyl-3-ammonio-1-propanesulfonate
*F*_r_: bilayer rupture force
FWHM: full width at half-maximum
GUVs: giant unilamellar vesicles
*h*: diffraction order
H: hexagonal phase
H_II_: inverted hexagonal phase
I_n_: integrated intensity of *n*^th^ order peaks of XRD measurements
*J*: curvature
L_α_: lamellar phase
O-Lyso-PC: 1-oleoyl-2-hydroxy-*sn*-glycero-3-phosphocholine
POPC: 1-palmitoyl-2-oleoyl-*sn*-glycero-3-phosphocholine
*q*: peak location
*Q*: scattering vector
**R_e_**: detergent–lipid ratio
**R**_**e**_^**sat**^: saturated detergent–lipid ratio
**R**_**e**_^**sol**^: solubilizing detergent–lipid ratio
RH: relative humidity
Rho B-DOPE: 1,2-dioleoyl-*sn*-glycero-3-phosphoethanolamine-*N*-(lissamine rhodamine B sulfonyl) (ammonium salt)
XRD: X-ray diffraction
