# Supplementary material for: Surfactant-Mediated Structural Modulations to Planar, Amphiphilic Multilamellar Stacks
Source: J Phys Chem B. 2023 Aug 16;127(34):7497–508. doi: 10.1021/acs.jpcb.3c01654 (PMC10476200; doi:10.1021/acs.jpcb.3c01654)
Supplement: Supplementary file 10 — jp3c01654_si_010.pdf [file jp3c01654_si_010.pdf]

SUPPORTING INFORMATION

Surfactant-Mediated Structural Modulations to Planar, Amphiphilic Multilamellar Stacks

Daniel J. Speer[a*], Marta Salvador-Castell[b*], Yuqi Huang[c*], Sunil K. Sinha[b], Atul N. Parikh[a,d]

[a] *Chemistry Graduate Group, University of California, Davis, One Shields Avenue, Davis, California, 95616*

[b] *Department of Physics, University of California, San Diego, 9500 Gilman Drive, La Jolla, California, 92093*

[c] *Department of Chemistry, University of California, Davis, One Shields Avenue, Davis, California, 95616*

[c] *Department of Biomedical Engineering, University of California, Davis, One Shields Avenue, Davis, California, 95616*

*Atul N. Parikh (anparikh@ucdavis.edu) and *Sunil K. Sinha (ssinha@physics.ucsd.edu)

Contents

Cover Page S1

Figure S1. POPC Multilamellar Stack Fluorescence Intensity S2

Figure S2. Indexed Q-Values of POPC and POPC:DDAPS Lamellar Mesophases S3

Figure S3. Surface Hydration of Dried POPC:Surfactant Mixtures S4

Figure S4. POPC:DDAPS Multilamellar Stack Fluorescence Intensity S5

Figure S5. POPC:DDAPS Multilamellar Stack Edge Fluorescence Intensity S6

Figure S6. Indexed Q-Values of POPC and POPC:O-Lyso-PC Lamellar Mesophases S7

Figure S7. POPC:O-Lyso-PC Multilamellar Stack Fluorescence Intensity S8

Figure S8. DDAPS And O-Lyso-PC Assemblies Upon Water Vapor Hydration S9

Video S1-S9 S10

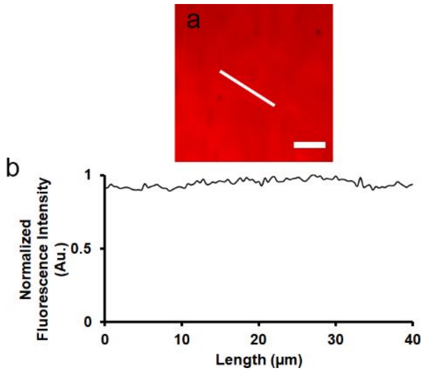

S1. POPC Multilamellar Stack Fluorescence Intensity. (a) A wide-field fluorescence microscopy image of a multilamellar mesophase assembled with POPC and 1 mol% Rho B-DOPE. The white line across the stack depicts the manually-drawn line along which fluorescence intensity of Rho B-DOPE was measured for (b). Scale bar, 20 μm. (c) The resulting plot of Rho B-DOPE fluorescence intensity along the line normalized to the background and maximum value.

Sample		q-values (Å^{-1})								
		q001	*q002*	*q003*	*q004*	*q005*	*q006*	*q007*	*q008*	*q009*
**POPC**		0.121	0.238	0.356	0.475	0.593	0.712	N.D.	N.D.	1.066
**POPC:DDAPS**	100:1	0.123	0.244	0.366	0.487	0.610	0.731	0.852	0.975	N.D.
	40:1	0.124	0.246	0.367	0.489	0.611	0.732	0.854	0.976	N.D.
	20:1	0.124	0.244	0.365	0.487	0.608	0.730	0.853	0.974	N.D.
	5:1	0.126	0.248	0.372	0.495	0.618	0.741	0.866	N.D.	N.D.
	5:2	0.128	0.251	0.377	0.502	0.629	0.754	0.879	N.D.	N.D.
	2:1	0.129	0.257	0.384	0.512	0.639	0.766	0.890	N.D.	N.D.
	**1:1**	0.135	0.267	0.399	0.532	0.666	N.D.	N.D.	N.D.	N.D.
	**2:3**	0.145	0.289	0.432	0.577	0.721	N.D.	N.D.	N.D.	N.D.
	**1:2**	0.145	0.291	0.436	0.581	0.729	N.D.	N.D.	N.D.	N.D.
	2:5	0.149	0.297	0.443	0.591	0.740	N.D.	N.D.	N.D.	N.D.
	1:3	0.138	0.276	0.412	0.549	N.D.	N.D.	N.D.	N.D.	N.D.
	**1:4**	0,14811	0.295	0.442	0.560	0.739	N.D.	N.D.	N.D.	N.D.

S2. Indexed Q-Values of POPC and POPC:DDAPS Lamellar Mesophases. Indexed q-values for POPC and POPC:DDAPS lamellar mesophases as measured by XRD experimentation. Molar ratios bolded here are plotted in **Figure 2**.

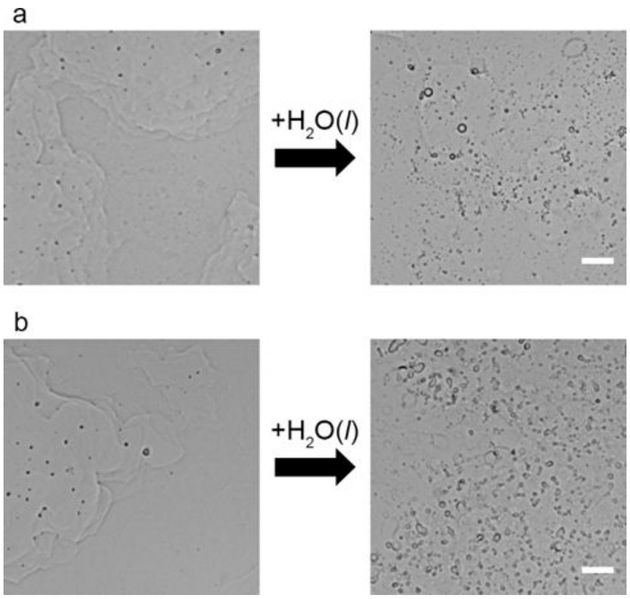

S3. Surface Hydration of Dried POPC:Surfactant Mixtures. (a) Images of a dried 1:1 POPC:DDAPS mixture pre- and post-exposure to 25 µL deionized water by surface contact on the left and right respectively. (b) Images of a dried 1:1 POPC:O-Lyso-PC mixture pre- and post-exposure to 25 µL deionized water by surface contact on the left and right respectively. No lamellar structures were formed post-exposure event for (a) and (b), instead lipidic, amorphous particulate matter was seen floating in the aqueous solution after dissolution.

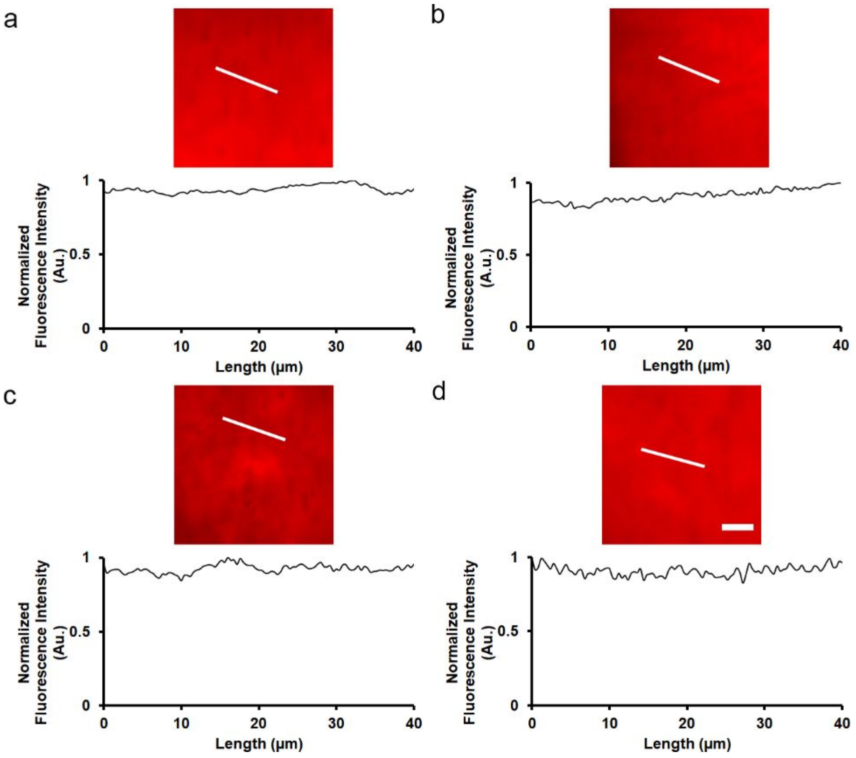

S4. POPC:DDAPS Multilamellar Stack Fluorescence Intensity. (a-d) The wide-field fluorescence microscopy images of multilamellar mesophases assembled using a POPC:DDAPS molar ratio of (a) 1:1, (b) 1:2, (c) 1:3, and (d) 1:4 with 1 mol% Rho B-DOPE. Scale bar, 20 μm. The white line across the images depicts the manually-drawn line along which fluorescence intensity of Rho B-DOPE was measured, with the resulting measurements of fluorescence intensity of the stack normalized to the background and maximum values plotted below each image for (a-d).

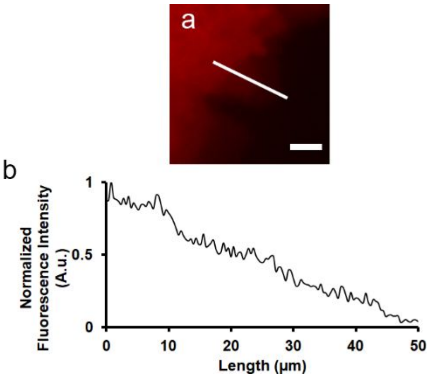

S5. POPC:DDAPS Mutilamellar Stack Edge Fluorescence Intensity. (a) The wide-field fluorescence microscopy image of an edge of an exemplary multilamellar mesophase assembled using a 1:2 molar ratio of POPC:DDAPS with a 1 mol% Rho B-DOPE dopant. Scale bar, 20 μm. The white line across the images depicts the manually-drawn line along which fluorescence intensity of Rho B-DOPE was measured, with the resulting measurements of fluorescence intensity of the stack normalized to the background and maximum values plotted below each image for (b).

Sample		q-values (Å^{-1})								
		q001	*q002*	*q003*	*q004*	*q005*	*q006*	*q007*	*q008*	*q009*
**POPC**		0.121	0.238	0.356	0.475	0.593	0.712	N.D.	N.D.	1.066
**POPC:O-Lyso-PC**	100:1	0.122	0.241	0.362	0.482	0.603	0.723	0.845	0.965	1.092
	40:1	0.123	0.242	0.362	0.483	0.603	0.723	0.844	0.964	1.081
	20:1	0.122	0.240	0.359	0.478	0.598	0.718	N.D.	N.D.	N.D.
	5:1	0.124	0.244	0.364	0.486	0.608	0.729	0.849	0.972	1.093
	5:2	0.125	0.246	0.369	0.492	0.615	0.737	N.D.	N.D.	N.D.
	2:1	0.122	0.239	0.358	0.477	0.597	N.D.	0.832	N.D.	N.D.
	**1:1**	0.122	0.239	0.357	0.476	0.595	0.714	N.D.	N.D.	N.D.
	**2:3**	0.127	0.251	0.376	0.499	0.624	N.D.	N.D.	N.D.	N.D.
	**1:2**	0.129	0.254	0.381	0.509	0.637	0.762	N.D.	N.D.	N.D.
	2:5	0.131	0.258	0.387	0.514	N.D.	N.D.	N.D.	N.D.	N.D.
	1:3	0.129	0.259	0.389	0.512	0.639	N.D.	N.D.	N.D.	N.D.
	**1:4**	0.132	0.260	0.389	0.519	0.649	N.D.	N.D.	N.D.	N.D.

S6. Indexed Q-Values of POPC and POPC:O-Lyso-PC Lamellar Mesophases. Indexed q-values for POPC and POPC:O-Lyso-PC lamellar mesophases as measured by XRD experimentation. Molar ratios bolded here are plotted in **Figure 6**.

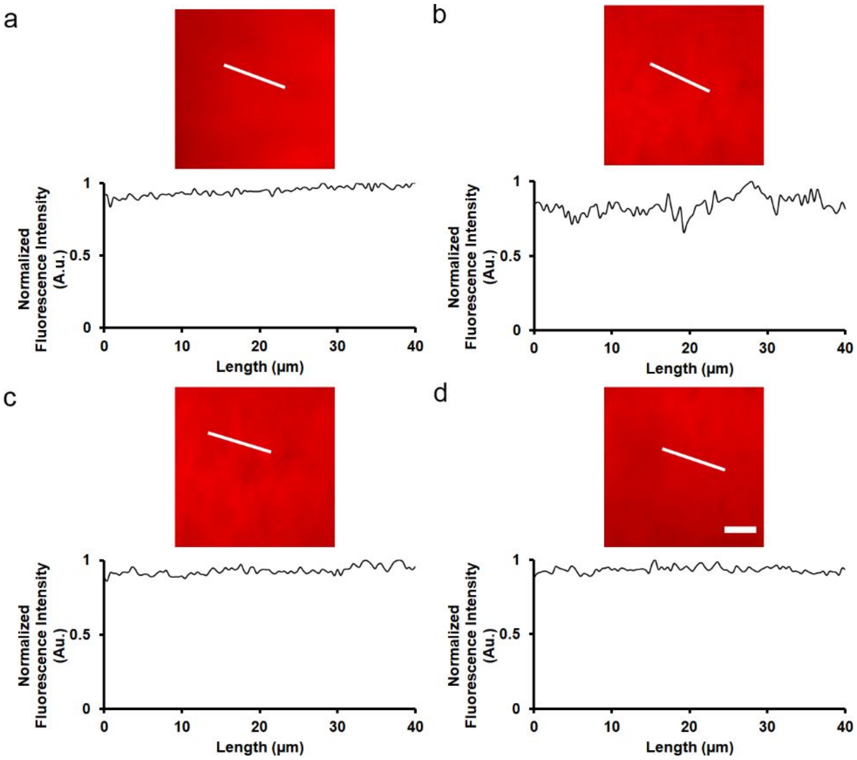

S7. POPC:O-Lyso-PC Multilamellar Stack Fluorescence Intensity. (a-d) The wide-field fluorescence microscopy images of multilamellar mesophases assembled using a POPC:O-Lyso-PC molar ratio of (a) 1:1, (b) 1:2, (c) 1:3, and (d) 1:4 with 1 mol% Rho B-DOPE. Scale bar, 20 μm. The white line across the images depicts the manually-drawn line along which fluorescence intensity of Rho B-DOPE was measured, with the resulting measurements of fluorescence intensity of the stack normalized to the background and maximum values plotted below each image for (a-d).

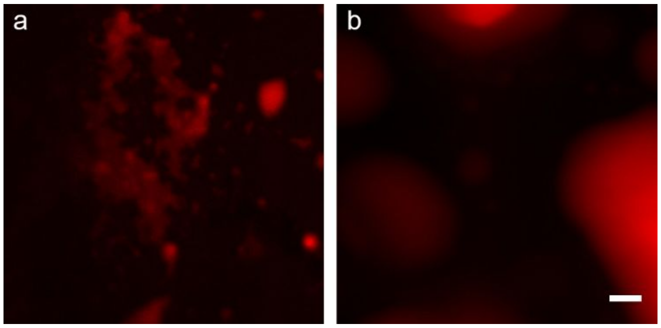

S8. DDAPS and O-Lyso-PC Assemblies Upon Water Vapor Hydration. (a) A wide-field fluorescence microscopy image of the morphologies assembled by a 0:1 molar ratio of POPC:DDAPS with 1 mol% Rho B-DOPE doped. (b) A wide-field fluorescence microscopy image of the morphologies assembled by a 0:1 molar ratio of POPC:O-Lyso-PC with 1 mol% Rho B-DOPE doped.

Video S1. Hydration of Dried POPC by Direct Contact with Liquid Water. Movie of dried POPC hydrated by 10 μL of deionized water aliquoted directly on top as visualized by brightfield microscopy. Scale bar, 20 μm.

Video S2. Hydration of Dried 1:1 POPC:DDAPS by Direct Contact with Liquid Water. Movie of dried 1:1 POPC:DDAPS mixture hydrated by 10 μL of deionized water aliquoted directly on top as visualized by brightfield microscopy. Scale bar, 20 μm.

Video S3. Hydration of Dried 1:2 POPC:DDAPS by Direct Contact with Liquid Water. Movie of dried 1:2 POPC:DDAPS mixture hydrated by 10 μL of deionized water aliquoted directly on top as visualized by brightfield microscopy. Scale bar, 20 μm.

Video S4. Hydration of Dried 1:3 POPC:DDAPS by Direct Contact with Liquid Water. Movie of dried 1:3 POPC:DDAPS mixture hydrated by 10 μL of deionized water aliquoted directly on top as visualized by brightfield microscopy. Scale bar, 20 μm.

Video S5. Hydration of Dried 1:4 POPC:DDAPS by Direct Contact with Liquid Water. Movie of dried 1:4 POPC:DDAPS mixture hydrated by 10 μL of deionized water aliquoted directly on top as visualized by brightfield microscopy. Scale bar, 20 μm.

Video S6. Hydration of Dried 1:1 POPC:O-Lyso-PC by Direct Contact with Liquid Water. Movie of dried 1:1 POPC:O-Lyso-PC mixture hydrated by 10 μL of deionized water aliquoted directly on top as visualized by brightfield microscopy. Scale bar, 20 μm.

Video S7. Hydration of Dried 1:2 POPC:O-Lyso-PC by Direct Contact with Liquid Water. Movie of dried 1:2 POPC:O-Lyso-PC mixture hydrated by 10 μL of deionized water aliquoted directly on top as visualized by brightfield microscopy. Scale bar, 20 μm.

Video S8. Hydration of Dried 1:3 POPC:O-Lyso-PC by Direct Contact with Liquid Water. Movie of dried 1:3 POPC:O-Lyso-PC mixture hydrated by 10 μL of deionized water aliquoted directly on top as visualized by brightfield microscopy. Scale bar, 20 μm.

Video S9. Hydration of Dried 1:4 POPC:O-Lyso-PC by Direct Contact with Liquid Water. Movie of dried 1:4 POPC:O-Lyso-PC mixture hydrated by 10 μL of deionized water aliquoted directly on top as visualized by brightfield microscopy. Scale bar, 20 μm.
